# Autophagy negatively regulates tumor cell proliferation through phosphorylation dependent degradation of the Notch1 intracellular domain

**DOI:** 10.18632/oncotarget.12986

**Published:** 2016-10-27

**Authors:** Ji-Seon Ahn, Eun-Jung Ann, Mi-Yeon Kim, Ji-Hye Yoon, Hye-Jin Lee, Eun-Hye Jo, Keesook Lee, Ji Shin Lee, Hee-Sae Park

**Affiliations:** ^1^ Hormone Research Center, School of Biological Sciences and Technology, Chonnam National University, Gwangju 61186, Republic of Korea; ^2^ Department of Pathology, Chonnam National University Medical School and Research Institute of Medical Sciences, Gwangju 61469, Republic of Korea

**Keywords:** autophagy, Notch1-IC, phosphorylation, degradation, tumorigenesis

## Abstract

Autophagy is a highly conserved mechanism that degrades long-lived proteins and dysfunctional organelles, and contributes to cell fate. In this study, autophagy attenuates Notch1 signaling by degrading the Notch1 intracellular domain (Notch1-IC). Nutrient-deprivation promotes Notch1-IC phosphorylation by MEKK1 and phosphorylated Notch1-IC is recognized by Fbw7 E3 ligase. The ubiquitination of Notch1-IC by Fbw7 is essential for the interaction between Notch1-IC and p62 and for the formation of aggregates. Inhibition of Notch1 signaling prevents the transformation of breast cancer cells, tumor progression, and metastasis. The expression of Notch1 and p62 is inversely correlated with Beclin1 expression in human breast cancer patients. These results show that autophagy inhibits Notch1 signaling by promoting Notch1-IC degradation and therefore plays a role in tumor suppression.

## INTRODUCTION

Notch1 is a type 1 single transmembrane receptor protein important for cell fate specification, differentiation in various systems and neuronal development such as neurogenesis and neural stem cell maintenance [1]. Notch1 signaling is aberrantly activated in breast cancer, and increased expression of the Notch1 intracellular domain (Notch1-IC) is associated with low survival rates in various cancers, including breast cancer [2–6]. Proliferation of cells derived from these cancers can be suppressed by pharmacological inhibition of Notch1. Therefore, preventing the generation of Notch1-IC is a potential strategy for treating various cancers [7, 8].

Genetic analysis of *Drosophila melanogaster* revealed a possible link between autophagy and the Notch1 signaling pathway involved in cell fate determination [9]. The mechanistic target of rapamycin (mTOR), a negative regulator of autophagy, activates Notch1 signaling [10]. The lack of autophagy triggers precocious activation of Notch1 signaling during Drosophila oogenesis, suggesting that autophagy suppresses Notch1 signaling [11]. However, the relationship between autophagy and Notch1 signaling in tumorigenesis and the precise regulatory mechanism is not well known. Many reports found that defective autophagy causes various cancers. *Beclin1* or *UVRAG* are monoallelically deleted in a high percentage of human breast and colon cancers respectively [12–14]. Atg5, a component of the ubiquitin-like protein conjugation systems, and Beclin1 have tumor suppressor effects in mouse xenograft models [12, 15]. It is clear these autophagy-related genes are involved in the regulation of tumorigenesis but it is not clear whether autophagy attenuates the tumorigenesis through the inhibition of oncogenic signal transduction.

In this study, we evaluated the crosstalk between autophagy and Notch1 signaling during tumorigenesis. We discovered that autophagic stimuli induced MEKK1 to phosphorylate the T2512 residue of Notch1-IC enabling its ubiquitination and degradation by Fbw7 ubiquitin ligase. We also found that the expression of Notch1 and Beclin1 protein in tissues of patients with breast cancer were negatively correlated. Notch1 inhibition significantly decreased growth, invasion, and tumorigenic activity of *Beclin1* knockdown cells. These data suggested that autophagy-induced MEKK1-mediated phosphorylation of Notch1-IC at the T2512 residue plays an important role in cancer prevention and could be a promising strategy to prevent cancer progression.

## RESULTS

### Autophagy attenuates Notch1 signaling

**Figure 1:**
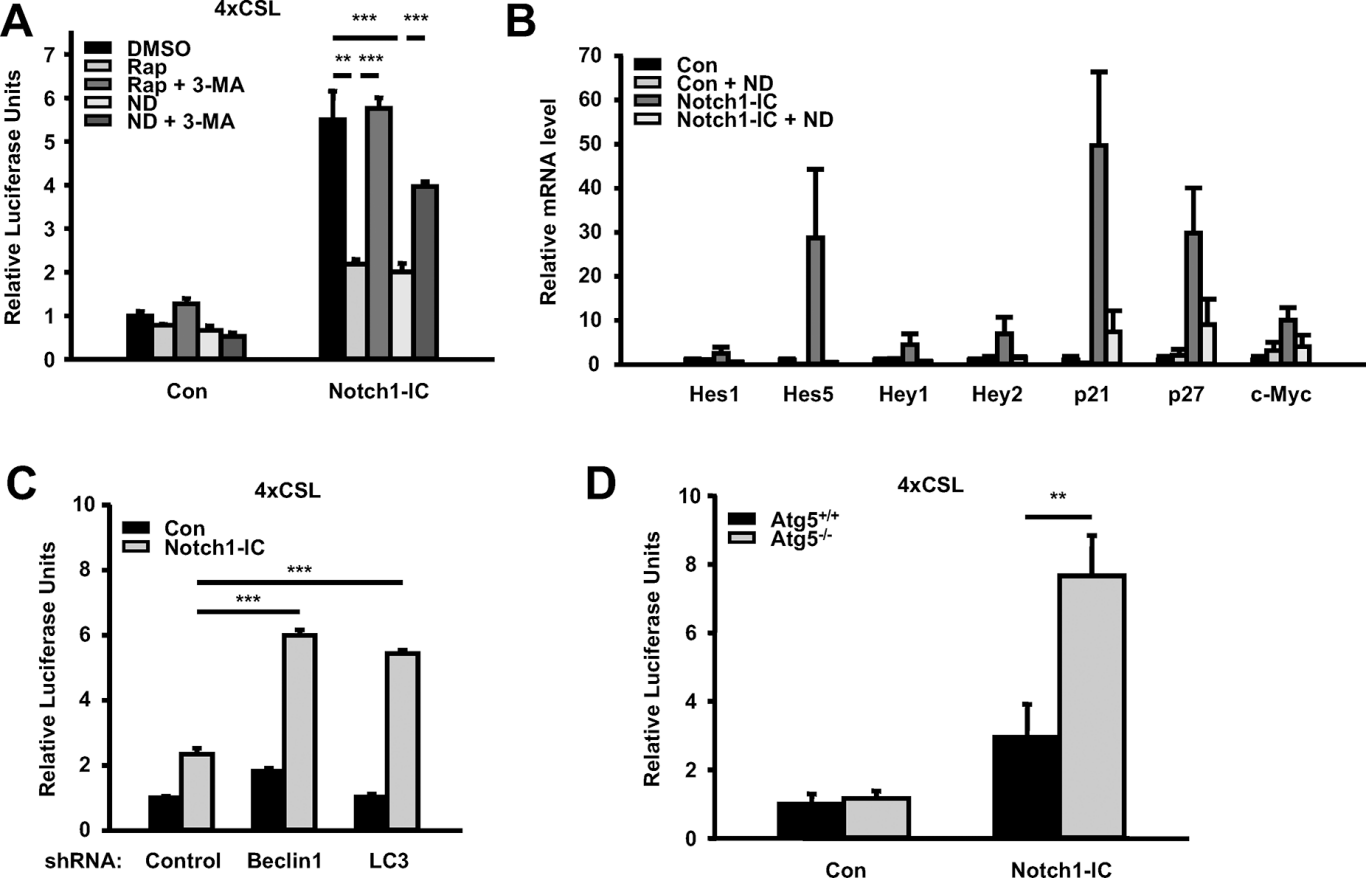
Autophagy attenuates Notch1 signaling (**A**) Rapamycin (Rap) treatment and nutrient deprivation attenuate the Notch1-IC transcriptional activity. HEK293 cells were transfected with the 4xCSL-Luc, together with pcDNA3 or Myc-Notch1-IC plasmids. After 48 h of transfection, the cells were treated with 2 μM rap, 10 mM 3-MA, or nutrient deprivation (ND) for 6 h, as indicated, and analyzed for Notch1-IC transcriptional activity (fold induction). (**B**) Nutrient deprivation reduces Notch1 target gene mRNA expression. HEK293 cells with pcDNA3 or Myc-Notch1-IC plasmids were starved for 4 hr. After RNA extraction and cDNA synthesis, quantitative RT-PCR was performed. (**C**) Knockdown of autophagy mediator *LC3*, *Beclin1*, and *p62* induce Notch1-IC transcriptional activity. HEK293 cells with pcDNA3 or Myc-Notch1-IC plasmids were transfected with shCon, shBeclin1, or shLC3 respectively. After transfection, the cells were analyzed for Notch1-IC transcriptional activity. (**D**) Knockdown of *Atg5* enhanced Notch1 signaling. *Atg5*^+/+^ and *Atg5*^−/−^ MEFs were transfected with the 4xCSL-Luc, together with pcDNA3 or Myc-Notch1-IC. After transfection, the cells were analyzed for Notch1-IC transcriptional activity. The relative luciferase activities were normalized with β-galactosidase activity. Data represent the mean ± SD from independent experiments performed in triplicate. ‘ND’ means nutrient-deprivation. ^**^p < 0.01; ^***^p < 0.001.

To understand the role of autophagy in Notch1 signaling, we treated HEK293 cells with rapamycin (rap) and cultured them in a nutrient-deprived medium. Rapamycin inhibits mTOR and induces autophagy. We found that both rapamycin and nutrient deprivation decreased the transcriptional activity of Notch1-IC. Whereas, inhibition of autophagy with 3-methyladenine (3-MA), the class III phosphoinositide 3-kinase inhibitor, rescued its activity (Figure 1A and Supplementary Figure S1A–S1C), supporting our premise that autophagy reduced the transcriptional activity of Notch1-IC. To determine whether autophagy-induced inhibition of Notch1 signaling decreases the transcriptional regulation of downstream Notch1 target genes (e.g., the *HES* family, the *HEY* family, *p21*, *p27*, and *c-Myc*), we measured the change in mRNA levels of *Hes1*, *Hes5*, *Hey1*, *Hey2*, *p21*, *p27*, and *c-Myc* by real-time quantitative PCR. The mRNA levels of Notch1 downstream targets decreased with the induction of autophagy by nutrient deprivation (Figure 1B), confirming that the expression of Notch1 target genes is suppressed by autophagy. Together, these results indicate that the induction of autophagy inhibits Notch1 signaling.

To further investigate whether autophagy suppresses Notch1 signaling, we performed luciferase reporter assays in autophagy defective HEK293 cells using shRNA knockdown. We found that a knockdown of the autophagy mediators, *LC3* or *Beclin1*, increased the transcriptional activity of Notch1-IC (Figure 1C). In contrast, overexpression of LC3 decreased the transcriptional activity and protein stability of Notch1-IC (Supplementary Figure S2A and S2B). Next, we used *Atg5*^+/+^ and *Atg5*^−/−^ MEFs, which differ in their ability to form autophagosomes [16]. We found that the transcriptional activity of Notch1-IC was significantly increased in *Atg5*^−/−^ MEFs compared with *Atg5*^+/+^ MEFs (Figure 1D), confirming that autophagy deficiency activates Notch1 signaling. Consequently, our results show that autophagy inhibits the Notch1 signaling pathway.

### Autophagy induces Notch1-IC degradation

**Figure 2:**
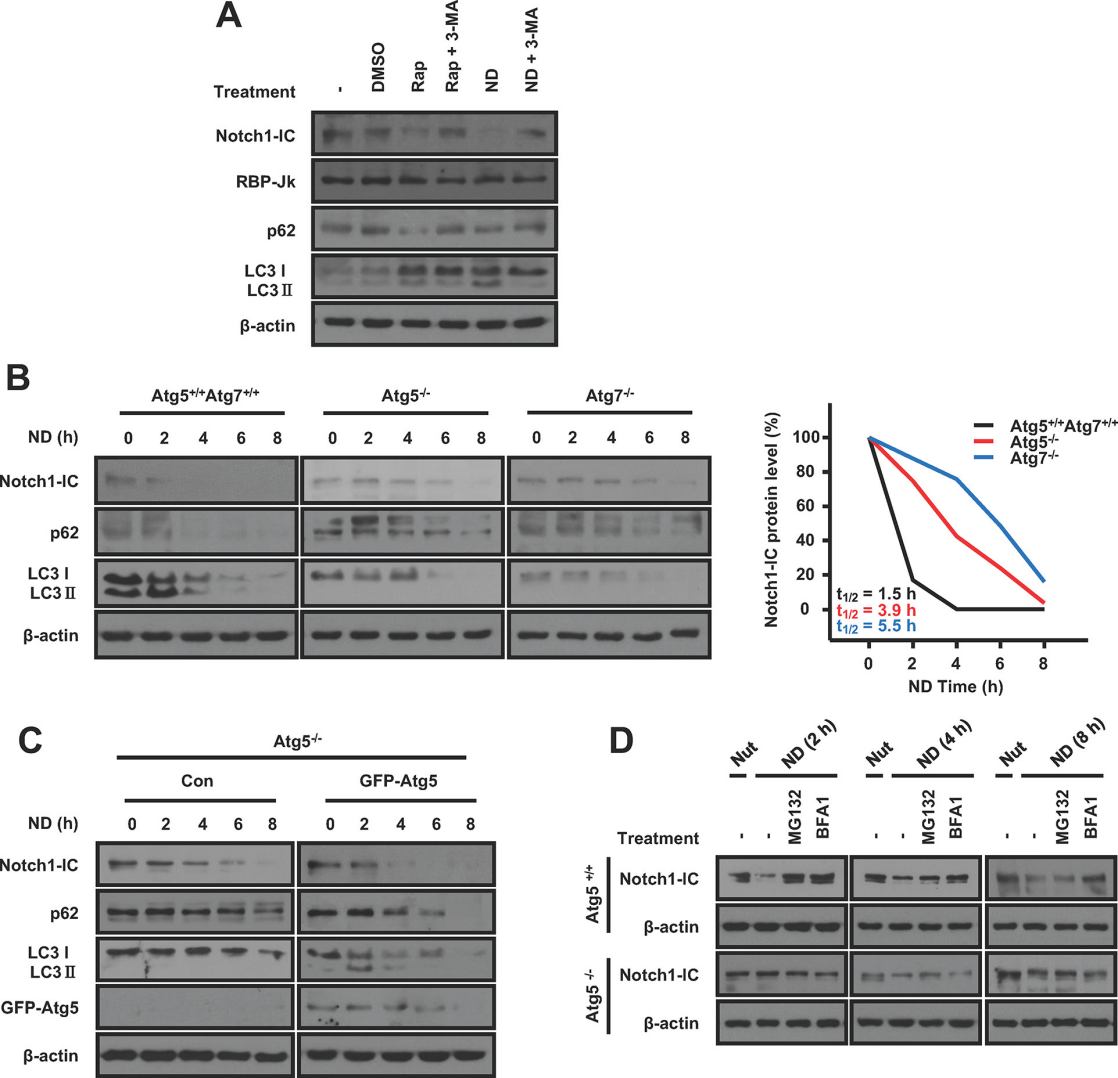
Autophagy induces Notch1-IC degradation (**A**) Notch1-IC is degraded by autophagy. HEK293 cells were treated with 2 μM rap or nutrient-deprivation medium. Each condition was reversed by treating 10 mM 3-MA, autophagy inhibitor. The cell lysates were subjected to immunoblotting. (**B**) Half-life of Notch1-IC was extended in *Atg5*^−/−^ and *Atg7*^−/−^ MEFs. WT and *Atg5*^−/−^ and *Atg7*^−/−^ MEFs were starved for the indicated durations and subjected to immunoblotting. Notch1-IC levels were quantified by ImageJ (Right panel). (**C**) *Atg5*^−/−^ MEFs with reintroduction of Atg5 promotes the Notch1-IC degradation. *Atg5*^−/−^ MEFs with pcDNA3 or GFP-Atg5 were starved for the indicated durations and subjected to immunoblotting. (**D**) The continued degradation of Notch1-IC in *Atg5*^−/−^ MEFs is proteasome-dependent. WT and *Atg5*^−/−^ MEFs were starved for 2, 4, or 8 h with 1 μM MG132 or 100 μM BFA1 to inhibit the different degradation systems and lysed for immunoblotting analysis. LC3 and p62 were used as autophagy markers. Results are representative of at least 3 independent experiments. ‘Nut’ means nutrient-rich. β-actin was used as a loading control.

To determine whether the autophagy pathway degrades the Notch1-IC, we treated HEK293 cells with rapamycin or incubated them in a nutrient-deprivation medium and analyzed the stability of the Notch1-IC protein. We found that autophagy induced by rapamycin or nutrient-deprivation resulted in a marked decrease in Notch1-IC protein levels. This was demonstrated by an increase in LC3 II and a decrease in p62, major substrates of autophagy. In accordance with this, the autophagy inhibitor 3-MA restored autophagy-induced Notch1-IC degradation (Figure 2A). However, the induction of autophagy had no effect on the level of the RBP-Jk protein, the DNA-binding transcriptional mediator of Notch1 signaling [17] (Figure 2A), confirming that autophagy selectively decreases the Notch1-IC protein levels. Atg5 and Atg7 are critical for the formation of autophagosomes [16, 18]. To validate our observations in autophagy deficient models, we analyzed the Notch1-IC protein levels in wild-type, *Atg5*^−/−^, and *Atg7*^−/−^ MEFs. We found that the half-life of Notch1-IC under nutrient-deprivation conditions was extended in *Atg5*^−/−^ and *Atg7*^−/−^ MEFs in comparison with wild-type MEFs (Figure 2B). In addition, we confirmed that the reintroduction of *Atg5* into *Atg5*^−/−^ MEFs, which restored the autophagy process, facilitated the decrease in Notch1-IC proteins (Figure 2C). These results suggest that the Notch1-IC is degraded by the autophagy pathway under nutrient-deprivation conditions.

Many reports showed that the Notch1-IC is degraded through the ubiquitin-proteasomal pathway [19, 20]. To investigate whether the Notch1-IC is degraded through the ubiquitin-proteasomal or autophagy-lysosomal pathway, we treated *Atg5*^+/+^ and *Atg5*^−/−^ MEFs with MG132, a proteasomal inhibitor, and bafilomycin A1 (BFA1), an autophagy-lysosomal inhibitor. Initially, Notch1-IC is degraded through the proteasome-dependent in *Atg5*^+/+^ cells. However, in *Atg5*^+/+^ MEFs, the longer the cells were starved, the more Notch1-IC proteins were degraded through the autophagy pathway (Figure 2D), confirming that the Notch1-IC is predominantly degraded by autophagy under nutrient deprivation conditions.

To further verify the degradation pathway of Notch1-IC under nutrient-rich conditions, we performed a western blot using MG132, the lysosomal inhibitor chloroquine and 3-MA, respectively. We confirmed that degradation of the Notch1-IC by LC3 decreased after MG132, chloroquine, and 3-MA treatment, confirming that degradation of the Notch1-IC by LC3 occurs through three pathways: proteasome-dependent, lysosome-dependent, and autophagy-dependent pathways (Supplementary Figure S3A–S3C). These data together suggest that autophagy inhibits Notch1 signaling by promoting Notch1-IC degradation.

### Nutrient-deprivation promotes the interaction between Notch1-IC and LC3, which is facilitated by the interaction between Notch1-IC and p62

**Figure 3:**
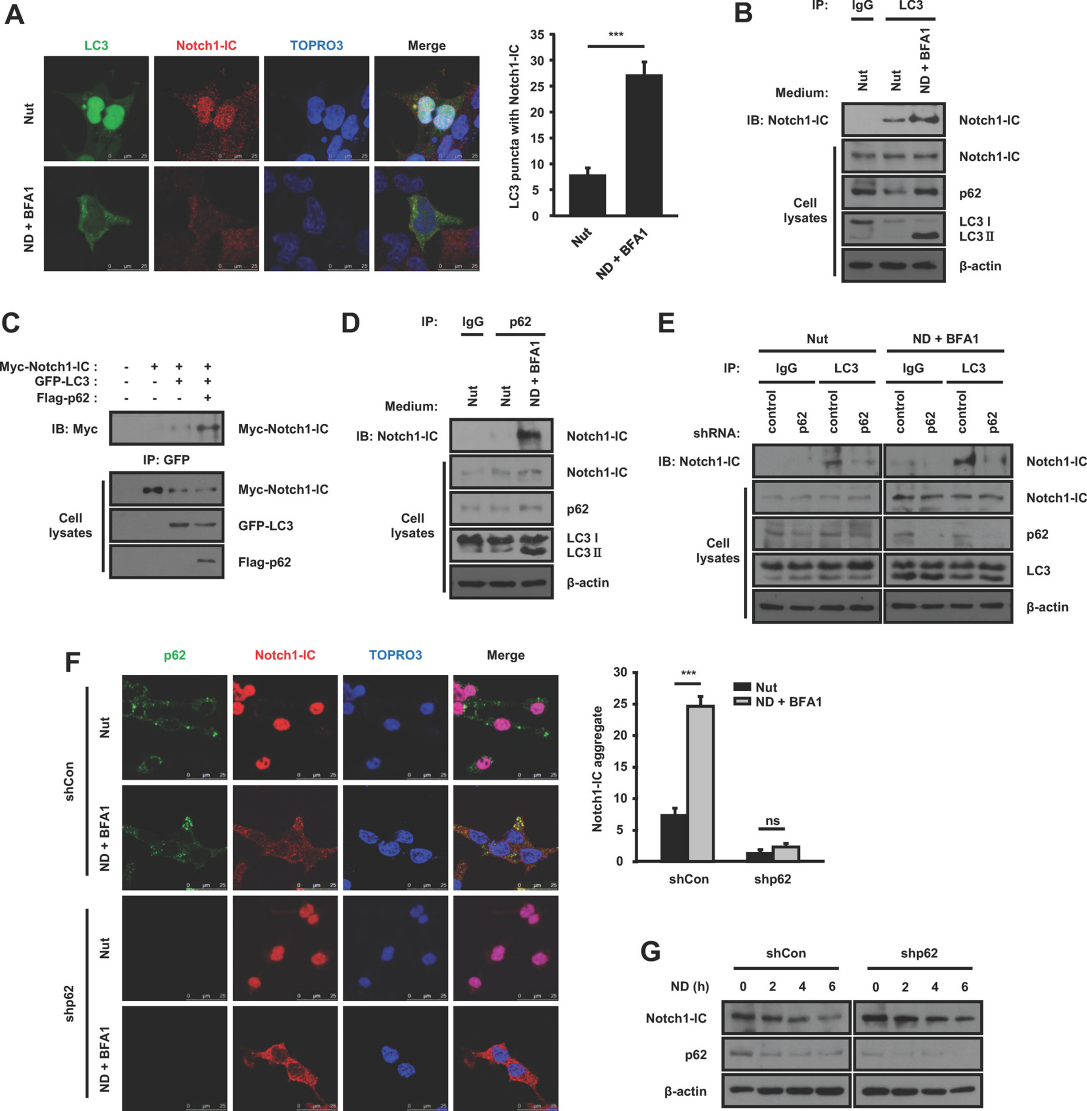
Nutrient-deprivation promotes the interaction between Notch1-IC and LC3, which is facilitated by the interaction between Notch1-IC with p62 (**A**) Localization of Notch1-IC and LC3 under nutrient-rich and nutrient-deprivation conditions. HEK293 cells with GFP-LC3 were cultured with 0.1 μM BFA1 under nutrient-rich and nutrient-deprivation conditions. The cells were stained with anti-Notch1-IC V1744 antibody and ToPro3. (**B**) Notch1-IC binds to LC3. Immunoprecipitates by anti-IgG control or anti-LC3 antibody from HEK293 cells cultured with BFA1 under nutrient-rich or nutrient-deprivation conditions were subjected to immunoblotting using anti-Notch1 antibody. (**C**) p62 promotes the interaction of Notch1-IC with LC3. Immunoprecipitates by anti-GFP antibody from HEK293 cells transfected with indicated plasmids were subjected to immunoblotting using anti-Myc antibody. (**D**) Notch1-IC binds to p62. Immunoprecipitates by anti-IgG control or anti-p62 antibody from HEK293 cells cultured with BFA1 under nutrient-rich or nutrient-deprivation conditions were subjected to immunoblotting using anti-Notch1 antibody. (**E**) Knockdown of *p62* decreases the interaction of Notch1-IC with LC3. HEK293 cells with shCon or shp62 were cultured with BFA1 under nutrient-rich or nutrient-deprivation conditions. Immunoprecipitates by anti-IgG control or anti-LC3 antibody from the cells were subjected to immunoblotting using anti-Notch1 antibody. (**F**) p62 is important for Notch1-IC aggregation and translocalization to autophagosome. HEK293 cells were transfected with shCon or shp62. The cells were cultured with BFA1 under nutrient-rich medium or nutrient-deprivation conditions and stained with anti-p62, anti-Notch1-IC V1744 antibodies, and ToPro3. (**G**) Half-life of Notch1-IC was extended by knockdown of *p62*. HEK293 cells with shCon or shp62 were starved for the indicated durations and subjected to immunoblotting. All images were confocal images of optical slice thickness ~1 μm. Scale bars represent 25 μm. Results are representative of at least 3 independent experiments. ^***^p < 0.001; ns: not significant (*p* > 0.05).

The previous report showed that LC3 may serve as a receptor to recruit proteins into autophagosomes [21]. To determine whether LC3 recruits the Notch1-IC into autophagosomes, we examined immunofluorescence staining and co-immunoprecipitation. Fluorescence staining revealed that clusters of LC3 dots mostly co-localized with Notch1-IC in puncta under nutrient-deprivation conditions, whereas LC3 was diffused over the control cells (Figure 3A). Notch1-IC bound to LC3 and this interaction was significantly increased under nutrient-deprivation conditions (Figure 3B). Previous reports showed that LC3 binds to tetrapeptide sequences, [W/F/Y]-X-X-[L/I/V], which have been found in several LC3-interacting proteins [22, 23]. However, Notch1-IC does not have an LC3-binding motif. We speculated that there is a protein linking Notch1-IC with LC3. Recent studies revealed that p62/SQSTM1 binds to polyubiquitinated protein aggregates and Atg8/LC3 on the autophagosome membrane to link target aggregates to autophagosomes for degradation [24–26]. To determine whether p62 is involved in the interaction between Notch1-IC and LC3, we performed co-immunoprecipitation experiments with and without overexpression of p62. We found that overexpression of p62 in HEK293 cells greatly increased the interaction between Notch1-IC and LC3 (Figure 3C). Furthermore, we found that endogenous Notch1-IC bound to p62 and this interaction increased greatly under nutrient-deprivation conditions (Figure 3D). These data support the notion that p62 is a mediator linking Notch1-IC to LC3. To confirm the effect of p62 on the interaction between Notch1-IC and LC3 in endogenous conditions, we introduced shp62 in HEK293 cells. We found that knockdown of *p62* reduced the Notch1-IC-LC3 interaction (Figure 3E). We hypothesized that p62 may facilitate the formation of Notch1-IC aggregates by self-oligomerization [22], leading to the localization of Notch1-IC into autophagosome. To investigate whether p62 affects the formation of endogenous Notch1-IC aggregates, we performed immunofluorescence staining using shp62. Nutrient deprivation induced co-localization of Notch1-IC and p62 in puncta into the cytoplasm (Figure 3F). In the *p62* knockdown cells, however, Notch1-IC were transferred to cytoplasm but not in puncta (Figure 3F), confirming that p62 is critical for Notch1-IC localization to the autophagosome. To determine whether the p62-dependent formation of Notch1-IC aggregates and localization to autophagosomes promote Notch1-IC degradation, we performed western blotting in the presence and absence of p62. Knockdown of *p62* inhibited the Notch1-IC turnover under nutrient-deprivation conditions (Figure 3G). These data together indicate that p62 facilitates Notch1-IC aggregation and interaction with LC3 to enable degradation through the autophagy pathway.

### Ubiquitination of Notch1-IC by Fbw7 is critical for the Notch1-IC-p62 interaction and degradation under nutrient-deprivation conditions

**Figure 4:**
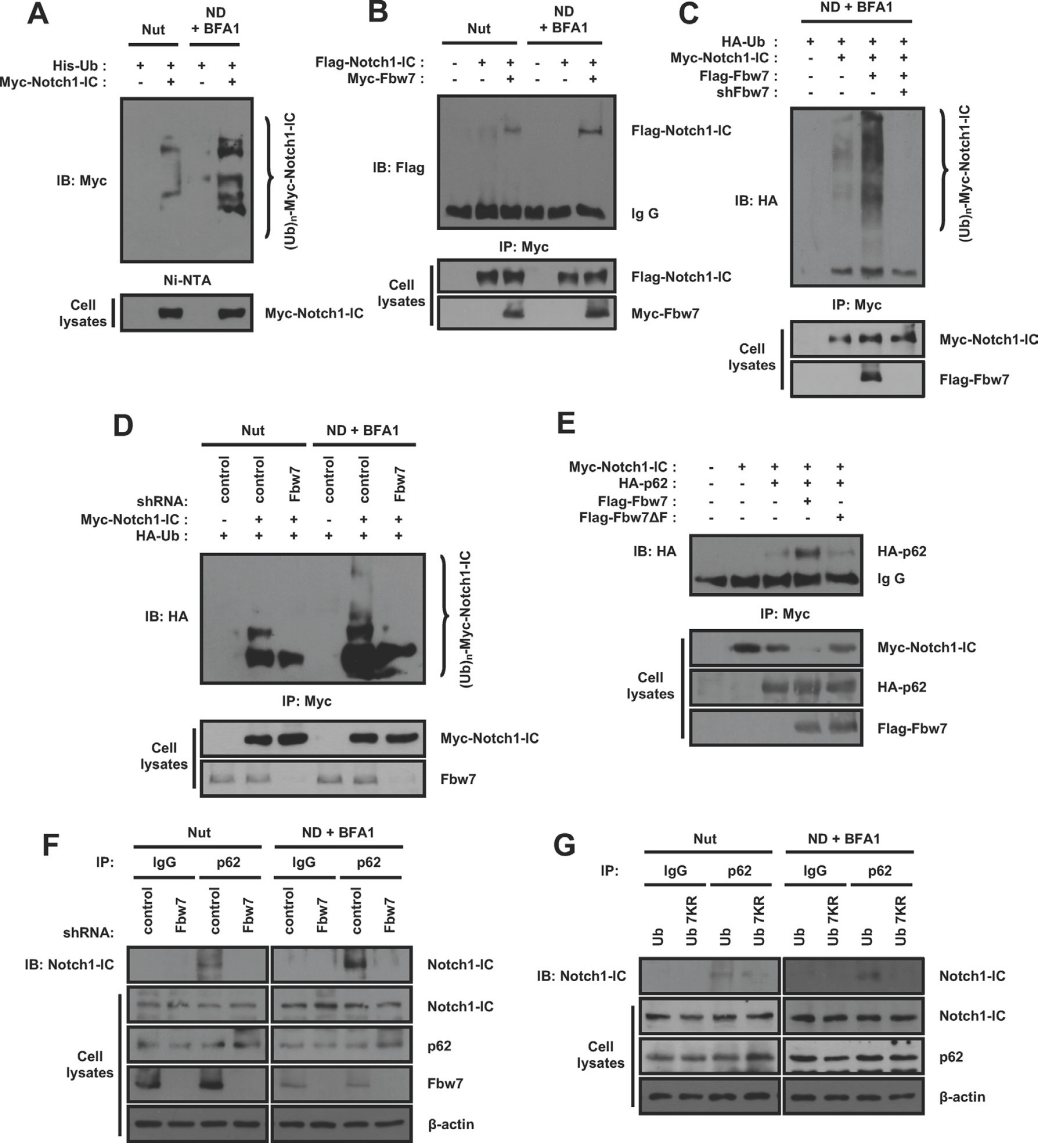
Ubiquitination of Notch1-IC under nutrient-deprivation condition by Fbw7 is critical for Notch1-IC-p62 interaction and degradation via autophagy (**A**) Nutrient-deprivation promotes ubiquitination of Notch1-IC. HEK293 cells transfected with indicated plasmids were cultured with BFA1 under nutrient-rich or nutrient-deprivation conditions. Cell lysates were precipitated by Ni-NTA and were subjected to immunoblotting using anti-Myc antibody. (**B**) Nutrient-deprivation promotes the interaction of Notch1-IC with Fbw7. HEK293 cells transfected with indicated plasmids were cultured with BFA1 under nutrient-rich or nutrient-deprivation conditions. Immunoprecipitates by anti-Myc antibody from the cells were subjected to immunoblotting using anti-Flag antibody. (**C**) Under nutrient-deprivation conditions, the ubiquitination of Notch1-IC is Fbw7-dependent. HEK293 cells transfected with indicated plasmids were cultured with BFA1 under nutrient-deprivation conditions. Immunoprecipitates by anti-Myc antibody from the cells were subjected to immunoblotting using anti-HA antibody. (**D**) Ubiquitination of Notch1-IC is decreased by shFbw7. HEK293 cells with shCon or shFbw7 were transfected with indicated plasmids. The cells were cultured with BFA1 under nutrient-rich or nutrient-deprivation conditions. Immunoprecipitates by anti-HA antibody from the cells were subjected to immunoblotting using anti-Myc antibody. (**E**) F-box domain in Fbw7 is required for Notch1-IC and p62 interaction. Immunoprecipitates by anti-Myc antibody from HEK293 cells transfected with indicated plasmids were subjected to immunoblotting using anti-HA antibody. (**F**) Knockdown of *Fbw7* decreases the interaction of Notch1-IC with p62. HEK293 cells with shCon or shFbw7 were cultured with BFA1 under nutrient-rich or nutrient-deprivation conditions. Immunoprecipitates by anti-IgG control or anti-p62 antibody from the cells were subjected to immunoblotting using anti-Notch1 antibody. (**G**) Polyubiquitination is required for Notch1-IC and p62 interaction. HEK293 cells with His-Ub or His-Ub-7KR were cultured with BFA1 under nutrient-rich or nutrient-deprivation conditions. Immunoprecipitates by anti-IgG control or anti-p62 antibody from the cells were subjected to immunoblotting using anti-Notch1 antibody. Results are representative of at least 3 independent experiments.

p62 recognizes the ubiquitinated proteins, forms aggregates by self-oligomerization and then binds to LC3 [27]. As a result, Notch1-IC needs to be ubiquitinated to interact with p62. To investigate whether Notch1-IC ubiquitination occurs in nutrient-deprivation conditions, we performed ubiquitination assays. We found that nutrient-deprivation promoted polyubiquitination of Notch1-IC (Figure 4A). Fbw7 is a well-known Notch1 and Notch1-IC E3 ubiquitin ligase, which ubiquitinates and degrades Notch1-IC via the proteasomal pathway [20, 28]. To determine the effect of nutrient-deprivation on the interaction between Notch1-IC and Fbw7, we performed co-immunoprecipitation assays. We found the interaction between Notch1-IC and Fbw7 increased under nutrient-deprivation conditions (Figure 4B). In addition, to determine the effect of Fbw7 on Notch1-IC ubiquitination, we used shRNA of Fbw7. We found that the ubiquitination of Notch1-IC was increased by overexpression of Fbw7, but decreased by *Fbw7* knockdown (Figure 4C). Further, nutrient deprivation enhanced Notch1-IC ubiquitination, and knockdown of Fbw7 resulted in a decrease in Notch1-IC ubiquitination (Figure 4D), confirming that autophagy-mediated Notch1-IC ubiquitination is Fbw7-dependent.

To investigate whether Fbw7 has any effects on the Notch1-IC-p62 interaction, we performed a co-immunoprecipitation assay. We found that Notch1-IC-p62 interaction increased when Fbw7 was overexpressed, but decreased in the presence of Fbw7 deleted F-box domain (Fbw7ΔF), a dominant negative mutant form of Fbw7 (Figure 4E). In addition, nutrient-deprivation elevated the interaction between Notch1-IC and p62, and this interaction was eliminated by *Fbw7* knockdown (Figure 4F), confirming that Fbw7 is important in enabling the interaction between Notch1-IC and p62. To confirm whether Fbw7-mediated Notch1-IC ubiquitination is crucial for the interaction between Notch1-IC and p62, we performed co-immunoprecipitation using ubiquitin mutant Ub-7KR (all seven lysines mutated to arginines), which is unable to form polyubiquitination chains. We found that a deficiency in polyubiquitination eliminated the Notch1-IC-p62 interaction (Figure 4G), verifying that polyubiquitination of Notch1-IC enables it to bind to p62. We also confirmed that the level of Notch1-IC was reduced by LC3 and Fbw7. This effect was reversed by Fbw7ΔF overexpression (Supplementary Figure S4A). Similarly, 3-MA restored the decreased levels of Notch1-IC caused by LC3 and Fbw7. (Supplementary Figure S4B). These data suggested that nutrient-deprivation promotes Notch1-IC ubiquitination by Fbw7, which enables the interaction between Notch1-IC and p62.

### Nutrient-deprivation induced MEKK1 phosphorylation facilitates the degradation of Notch1-IC

**Figure 5:**
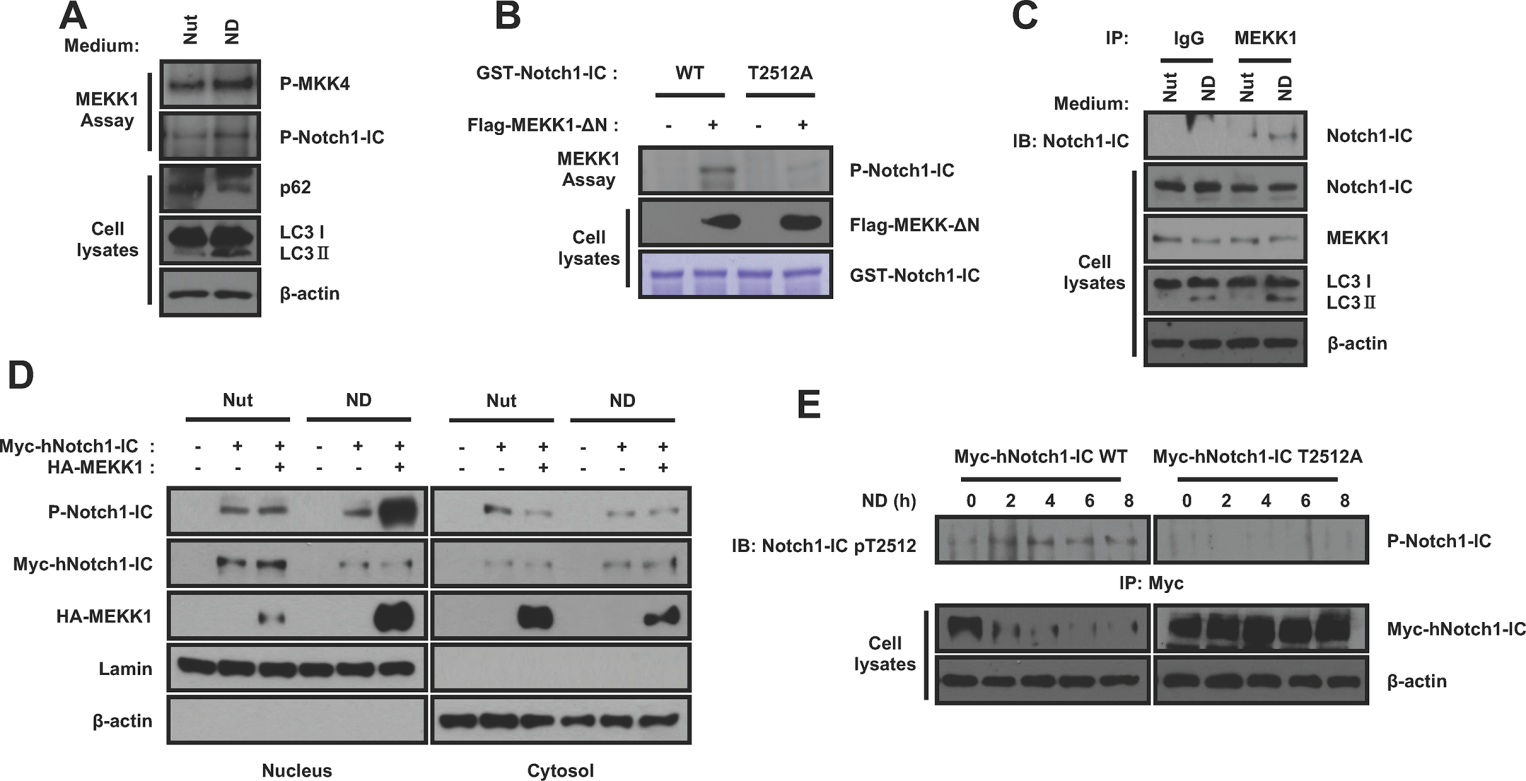

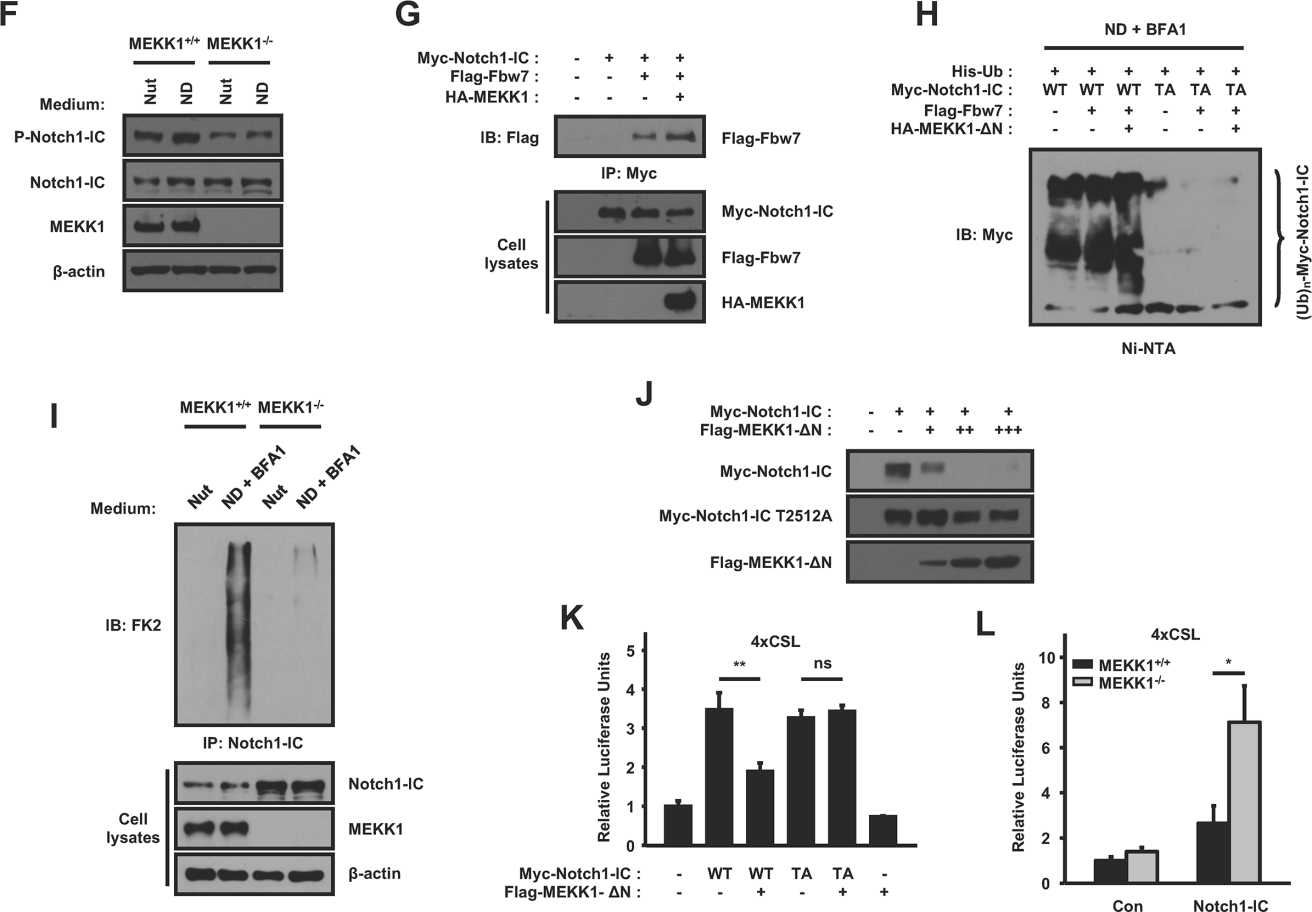
Notch1-IC phosphorylation by MEKK1 is critical for the regulation of Notch1-IC stability during nutrient-deprivation (**A**) Nutrient-deprivation induces MEKK1 activation. HEK293 cells with HA-MEKK1 were cultured with BFA1 under nutrient-rich or nutrient-deprivation conditions. Immunoprecipitates by anti-HA antibody from the cells were subjected to *in vitro* kinase assays with GST-MKK4 or GST-Notch1-IC as substrates. (**B**) MEKK1 phosphorylates Notch1-IC T2512. Immunoprecipitates by anti-Flag antibody from the cells with pcDNA3 or Flag-MEKK1-ΔN were subjected to *in vitro* kinase assays with GST-Notch1-IC WT and GST-Notch1-IC T2512A mutant as substrates. (**C**) Nutrient-deprivation induces the interaction of Notch1-IC with MEKK1. HEK293 cells were cultured with BFA1 under nutrient-rich or nutrient-deprivation conditions. Immunoprecipitates by anti-IgG control or anti-MEKK1 antibody from the cells were subjected to immunoblotting using anti-Notch1 antibody. (**D**) Nutrient-deprivation induces the MEKK1 nuclear localization and MEKK1-dependent Notch1-IC phosphorylation. HEK293 cells were fractionated into cytosolic and nuclear fractions. Each fraction was subjected to immunoblotting using anti-Notch1-IC pT2512, anti-Notch1-IC, anti-MEKK1, anti-Lamin B, and anti-β-actin antibodies. (**E**) Nutrient-deprivation induces the Notch1-IC T2512. HEK293 cells with Myc-hNotch1-IC WT or T2512A mutant were starved for the indicated durations. Immunoprecipitates by anti-Myc antibody from the cells were subjected to immunoblotting using anti-Notch1-IC pT2512 antibody. (**F**) MEKK1 is important to phosphorylate the Notch1-IC T2512 under nutrient-deprivation conditions. *MEKK1*^+/+^ and *MEKK1*^−/−^ MEFs were cultured with BFA1 under nutrient-rich or nutrient-deprivation conditions and subjected to immunoblotting using anti-Notch1-IC pT2512, anti-Notch1-IC, anti-MEKK1, and anti-β-actin antibodies. (**G**) MEKK1 induces the interaction of Notch1-IC with Fbw7. Immunoprecipitates by anti-Myc antibody from HEK293 cells transfected with indicated plasmids were subjected to immunoblotting using anti-Flag antibody. (**H**) MEKK1 induces the ubiquitination of Notch1-IC by Fbw7. HEK293 cells transfected with indicated plasmids were cultured with BFA1 under nutrient-deprivation conditions. Cell lysates were precipitated by Ni-NTA and were subjected to immunoblotting using anti-Myc antibody. (**I**) Ubituitination of endogenous Notch1-IC is decreased in *MEKK1*^−/−^ MEFs. *MEKK1*^+/+^ and *MEKK1*^−/−^ MEFs were cultured with BFA1 under nutrient-rich or nutrient-deprivation conditions. Cell lysates were precipitated by anti-Notch1-IC antibody and were subjected to immunoblotting using anti-FK2 antibody. (**J**) MEKK1 decreases the Notch1-IC protein stability. HEK293 cells were transfected with indicated plasmids and subjected to immunoblotting using anti-Myc and anti-Flag antibodies. (**K**) and (**L**) MEKK1 inhibits the Notch1-IC transcriptional activity. Lysates of HEK293 cells transfected with Flag-MEKK1-ΔN and Myc-hNotch1-IC WT or T2512A mutant (K) and *MEKK1*^+/+^ and *MEKK1*^−/−^ MEFs transfected with pcDNA3 or Myc-Notch1-IC (L) were analyzed for Notch1-IC luciferase reporter assay. The relative luciferase activities were normalized with β-galactosidase activity. Results are representative of at least 3 independent experiments. Data represent means ± SD from independent experiments performed in triplicate. ^*^p < 0.05; ^**^p < 0.01; ns: not significant (*p* > 0.05).

Recent studies showed that the phosphorylation of the Notch1-IC Threonine 2512 (T2512) residue is essential for the ubiquitination and degradation of Notch1-IC by Fbw7 [29–31]. From our kinase screening, we found that MEKK1 phosphorylates Notch1-IC. To determine whether MEKK1 affects autophagy-induced Notch1-IC degradation, we performed *in vitro* kinase assay. We confirmed that MEKK1 was activated under nutrient-deprivation conditions by demonstrating an increase in MKK4 phosphorylation (Figure 5A) and in the mutant active form of MEKK1 (MEKK1-ΔN) phosphorylated wild-type Notch1-IC but not in the Notch1-IC mutant Notch1-IC T2512A substituted threonine 2512 to alanine (Figure 5A and 5B). This confirms that MEKK1 phosphorylates Notch1-IC at the T2512 site. To confirm the physical interaction between Notch1-IC and MEKK1, we performed co-immunoprecipitations. We found that MEKK1 bound to Notch1-IC and that this interaction was increased under nutrient-deprivation conditions (Figure 5C). To determine whether MEKK1 phosphorylates nuclear or cytosolic Notch1-IC, we performed subcellular fractionation. We found that nutrient-deprivation promoted the MEKK1 nuclear translocation and phosphorylation of nuclear Notch1-IC. (Figure 5D). In addition, nutrient-deprivation induced the phosphorylation of Notch1-IC, but not at the Notch1-IC T2512A residue (Figure 5E). To investigate whether the phosphorylation of Notch1-IC was due to MEKK1, we used *MEKK*^+/+^ and *MEKK1*^−/−^ MEFs. We found that nutrient-deprivation induced Notch1-IC phosphorylation at the T2512 residue in *MEKK1*^+/+^ MEFs, but not in *MEKK1*^−/−^ MEFs (Figure 5F). These data suggest that MEKK1 is crucial for nutrient-deprivation induced Notch1-IC phosphorylation.

To investigate whether phosphorylation of Notch1-IC by MEKK1 increases the interaction between Notch1-IC and Fbw7, we performed co-immunoprecipitation assays. We found that the Notch1-IC-Fbw7 interaction was increased by MEKK1 overexpression (Figure 5G). To determine whether MEKK1 increases Fbw7-induced Notch1-IC ubiquitination, we performed ubiquitination assays. MEKK1 increased the Notch1-IC ubiquitination in Fbw7 dependent manner (Figure 5H). We also found that endogenous Notch1-IC ubiquitination was decreased in *MEKK1*^−/−^ cells compared to *MEKK1*^+/+^ cells (Figure 5I). In accordance with this, we confirmed that the degradation of Notch1-IC was increased by MEKK1 (Figure 5J). However, MEKK1 had no effect on the ubiquitination and degradation of the Notch1-IC T2512A residue (Figure 5H and 5J). The transcriptional activity of Notch1-IC, but not Notch1-IC T2512, was also decreased by MEKK1 (Figure 5K). Furthermore, the transcriptional activity of Notch1-IC was increased in MEKK1^−/−^ cells compared to *MEKK1*^+/+^ cells (Figure 5L). Taken together, these data indicate that nutrient-deprivation induced MEKK1 to phosphorylate Notch1-IC on the T2512 residue and thereby promote Notch1-IC ubiquitination and degradation.

### Negative correlation between Notch1-IC and autophagy in human breast cancer

**Figure 6:**
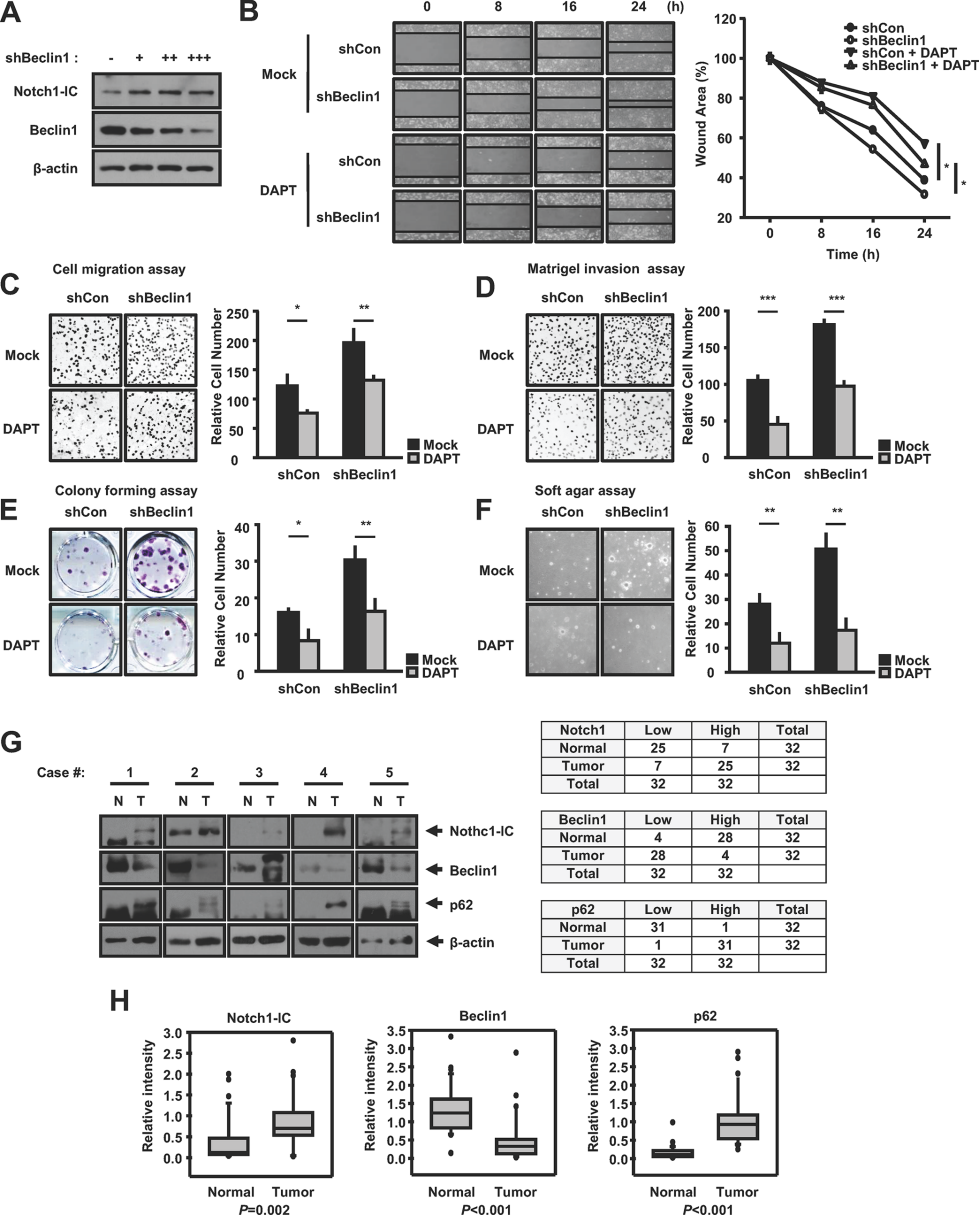
Negative correlation between Notch1-IC and autophagy in breast cancer (**A**) Notch1-IC protein stability is increased by knockdown of *Beclin1.* HEK293 cells were transfected with shBeclin1 in dose-dependent manner and subjected to immunoblotting using anti-Notch1-IC, anti-Beclin1, and anti-β-actin antibodies. (**B**–**E**) MDA-MB-231 cells stably expressing shCon or shBeclin1 were treated with or without 2 μM DAPT. B. Knockdown of *Beclin1* induces wound-healing migration ability. The wound closure was quantified for the indicated durations and was analyzed by measuring the width of the remaining unmigrated area. Wound area is plotted (right). C. Knockdown of *Beclin1* induces cell migration ability. The cells were seeded onto fibronectin-coated Transwell inserts. The migrating cells were stained by DAPI and quantified by counting. The number of migrated cells is plotted (right). D. Knockdown of *Beclin1* induces cell invasion ability. The cells were seeded onto a Matrigel invasion chamber. The invading cells were stained by DAPI and quantified by counting. The number of migrated cells is plotted (right). E. Knockdown of *Beclin1* induces the ability of the cells to form anchorage-dependent colonies. The cells were seeded onto 24 well plates at a density of 100 cells per well and were incubated for 10 days. The cell colonies were stained with crystal violet. The graph shows the number of colonies formed by each cell. (**F**) Knockdown of *Beclin1* induces the ability of the cells to form anchorage-independent colonies. The cells were plated at a density of 5 × 10^3^ cells in a top medium containing 0.3% agarose and were incubated for 10 days. The cell colonies were stained with crystal violet. The graph shows the number of colonies formed by each cell. (**G**) and (**H**) Negative correlation of Notch1-IC and autophagy in human breast cancer samples versus normal tissues. (G) Lysates of paired human normal and breast cancer tissues were subjected to immunoblotting using anti-Notch1, anti-Beclin1, anti-p62, and anti-β-actin antibodies. (H) Ranking plot of each protein in normal and breast tumor tissues. The difference in Notch1-IC, Beclin1 and p62 between normal and tumor tissue were examined by Wilcoxon Signed-Rank test. Results are representative of at least 3 independent experiments. Data represent means ± SD from independent experiments performed in triplicate. ^*^p < 0.05; ^**^p < 0.01; ^***^p < 0.001.

To explore the effects of autophagy-induced Notch1-IC degradation in cancer, we examined the correlation between Notch1-IC and autophagy in breast tumor. To investigate further the autophagic regulation of Notch1 signaling, we generated a stable *Beclin1* knockdown MDA-MB-231 breast cancer cell line. We confirmed that knockdown of beclin1 increased the Notch1-IC protein stability (Figure 6A). As Notch1 signaling is improved in wound healing [32], we performed a wound healing assay. The wound healing assay showed that the migratory ability of *Beclin1* knockdown cells were greater than that of control cells (Figure 6B). Recent studies showed that Notch1 activation stimulates the migration of breast cancer cells [33] and Notch1 knockdown suppresses proliferation, migration and metastasis [34]. To investigate whether autophagy affects Notch1-induced migration ability of breast cancer cells, we performed migration and invasion assays. We found that the migration and invasion ability of *Beclin1* knockdown cells had increased (Figure 6C and 6D). In addition, *Beclin1* knockdown cells showed increased anchorage-dependent and anchorage-independent cell growth (Figure 6E and 6F). The gamma-secretase inhibitor DAPT (N-[N-(3,5-Difluorophenacetyl)-L-alanyl]-S-phenylglycine t-butyl ester), which inhibits Notch1 signaling, decreased the ability of *Beclin1*-knockdown cells to migrate, invade, and form colonies (Figure 6B–6F). Together, these data indicate that autophagy inhibits Notch1-IC-induced cancer cell migration and tumorigenesis.

To further examine the relationship between autophagy and Notch1-IC, we examined the expression of Notch1-IC, Beclin1, and p62 in 31 freshly prepared human breast tumor specimens and matching normal breast tissue samples by western blotting. The negative correlation between Notch1-IC and Beclin1 and the positive correlation between Notch1-IC and p62 were observed in human breast tumor and its adjacent normal tissues (Figure 6G and 6H). Taken together, impairment of autophagy may contribute to the aberrant activation of Notch1 and it might be associated with tumorigenesis.

## DISCUSSION

**Figure 7:**
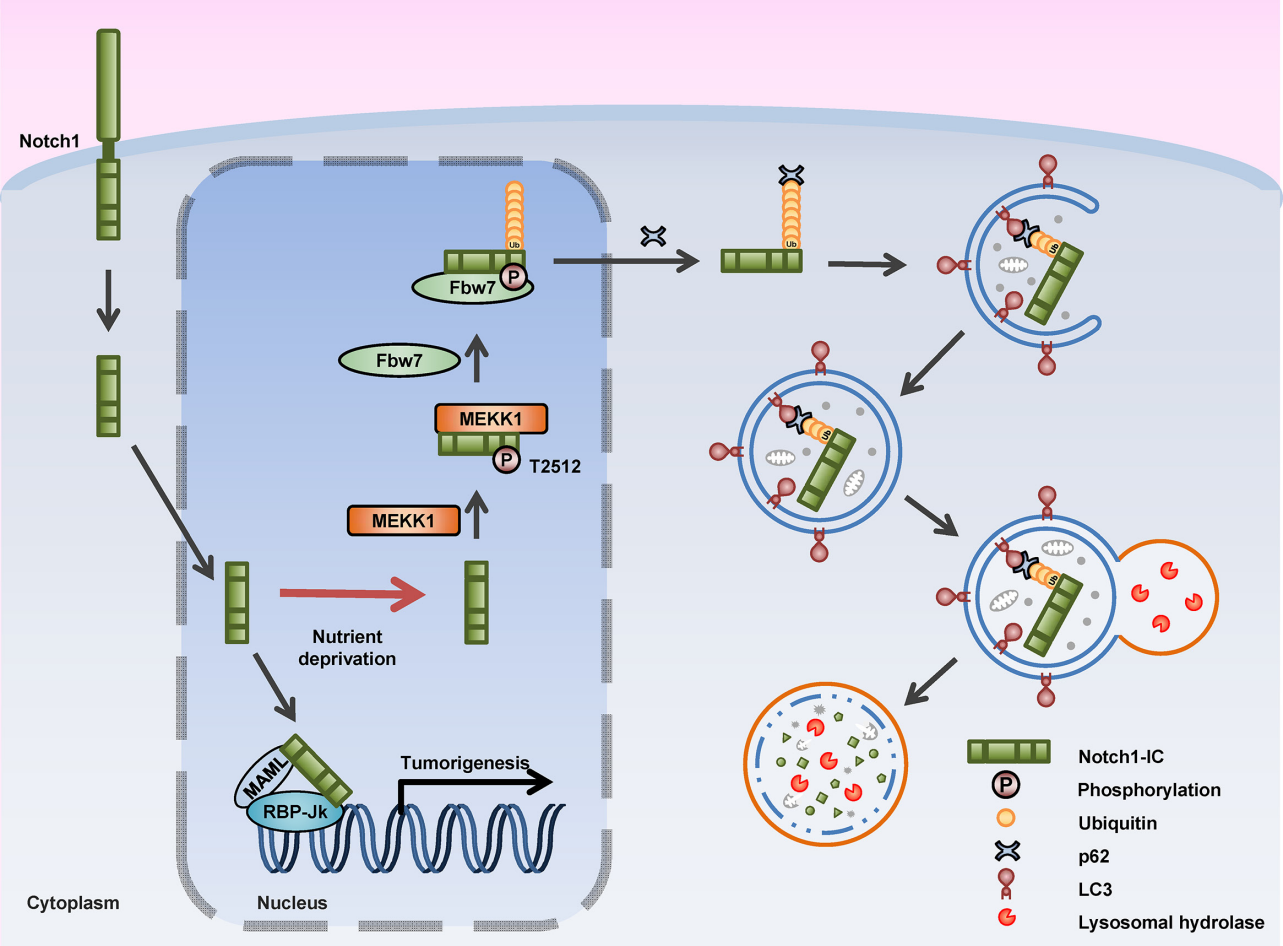
Schematic Model for phosphorylation, ubiquitination, and degradation of Notch1-IC in autophagy The diagram depicts autophagy-mediated inhibition of Notch1 signaling. The canonical Notch1 signaling pathway is initiated when the ligand in intact cells binds to the Notch1 receptor. Then, Notch1 is cleaved by γ-secretase forming Notch1-IC, which translocates into the nucleus. The Notch1-IC acts as a transcription factor promoting Notch1-IC target gene expression. Under nutrient-deprivation condition, Notch1-IC in the nucleus is phosphorylated by MEKK1 at the T2512 residue, ubiquitinated by Fbw7 and translocalized to the cytosol. Then, p62 binds to and forms aggregates with the ubiquitinated Notch1-IC. LC3 binds to p62 and leads to the formation of the autophagosome. Lysosomes combine with the autophagosome and then Notch1-IC is degraded.

In this study, we showed that autophagy promotes the degradation of the Notch1 intracellular domain via the phosphorylation of the T2512 residue by MEKK1, and therefore suppresses tumor cell growth and migration (Figure 7). The finds from the present study indicated that upregulation and downregulation of Notch1-IC and Beclin1 expression, respectively, were inversely correlated in patients with breast cancer. Aberrant activation of Notch1 signaling produces Notch1-IC in various cancers, including breast cancer [5, 35, 36]. Therefore, it was suggested that an anticancer drug involving small molecule inhibitors aimed at downregulating Notch1-IC production should be developed [7, 8].

We used the nutrient deprivation of cells to induce the autophagy. When the cells are in nutrient deprivation conditions, autophagy is often induced to reduce or arrest cell growth [37]. Autophagy is known to impair mTOR-dependent cell growth through selective degradation of p62 [38]. Therefore, we assumed that autophagy might regulate other cell growth-regulating proteins. Notch signaling pathway promotes cell growth and proliferation [39]. A recent study shows that AMP-activated protein kinase (AMPK), known as the energy sensor and upstream of autophagy, regulates Notch signaling through mTORC1 under influence of nutrient status [40]. Thus, nutrient deprivation negatively regulates Notch signaling to reduce cell growth.

Phosphorylation of the Notch1-IC T2512 residue is important for its recognition by Fbw7, which facilitates its degradation through the proteasome [29–31]. However, the kinase that phosphorylates the T2512 residue has not yet been definitively determined. We performed the screening to identify the novel kinase that phosphorylated the Notch1-IC T2512 residue using a phospho-T2512 specific antibody. We found that MEKK1 is potential candidates for the phosphorylation of the Notch1-IC T2512 residue. Many studies have shown the relationship between MAPK signaling with Notch1 signaling [41–45], however, whether the MEKK1 is associated with Notch1 signaling is completely unknown. We confirmed that MEKK1 binds to Notch1-IC and phosphorylates the Notch1-IC T2512 residue. This phosphorylation is increased under nutrient-deprivation conditions. MEKK1 promotes the interaction between Notch1-IC and Fbw7 and thus the polyubiquitination and degradation of Notch1-IC. As a result, MEKK1 suppresses the Notch1-IC protein stability and transcriptional activity.

We also found that autophagy inhibits Notch1-IC-induced tumorigenesis. Additionally, autophagy is inversely correlated with Notch1-IC expression and activation of Notch1 signaling in human breast tumors. A recent study showed that T-ALL cell growth is suppressed by simultaneous treatment with the mTOR inhibitor rapamycin and the gamma secretase inhibitor GSI [46]. Our research suggests that autophagy functions as a tumor suppressor by suppressing Notch1 signaling in breast cancer, however, additional studies are required to explore the effect of autophagy on other oncogenic pathways in different cancers.

Our finding demonstrates how autophagy regulates Notch1 signaling. Under nutrient-deprivation condition, MEKK1 phosphorylates the Notch1-IC T2512 residue enabling Fbw7 to recognize and ubiquitinate the phosphorylated Notch1-IC. Ubiquitinated Notch1-IC binds to p62. p62 acts as a bridge between Notch1-IC and LC3 in the autophagosome enabling the autophagosome to engulf them and undergo autophagic degradation. In this way, autophagy decreases Notch1 signaling-dependent tumorigenesis. However, whether autophagy regulates other oncogenic signaling pathways and other Notch1 functions like stem cell differentiation remains to be discerned.

## MATERIALS AND METHODS

### Antibodies and reagents

The following antibodies were used for immunoblotting, immunoprecipitation, and immunofluorescence staining: Anti-p62 (sc-25575), anti-Notch1 (sc-6014), anti-Fbw7 (sc-21185), anti-Beclin1 (sc-11427), anti-RBP-Jk (sc-28713), anti-β-actin (sc-47778), anti-GFP (sc-8334), anti-MEKK1 (sc-252), normal rabbit IgG (sc-2027), and normal goat IgG (sc-2028) were purchased from Santa Cruz biotechnology. Anti-LC3 (NB100-2220) was purchased from Novus Biologicals. Anti-cleaved Notch1 (Val1744) was purchased from Cell Signaling. Anti-Flag (A2220) was purchased from Sigma. Anti-phospho-ser/thr-pro was purchased from Upstate Biotechnology. Anti-Myc (9E10) and anti-HA (12CA5) were purified by agarose resin. Rapamycin and MG132 were obtained from Cayman Chemical and 3-MA from Acros Organics. BFA1 was obtained from Wako Reagent. DAPT and Chloroquine were purchased from Sigma.

### RNA interference

ShRNAs were purchased from Shanghai GenePharma. The double-stranded oligonucleotides targeting specific genes were cloned into pGPU6/Neo vectors. The sequences of the oligonucleotides are as follows: Beclin1, 5′-CAGTTTGGCACAATCAATA-3′, LC3, 5′-CATGAGCGAGTTGGTCAAGAT-3′, p62, 5′-GCATTGAAGTTGATATCGAT-3′, and Fbw7, 5′-CCTT CTCTGGAGA GAGAAA-3′. The control shRNA sequence was 5′-TTCTCCGAACGTGTCACGT-3′.

### Cell culture and transfection

HEK293 cells were maintained in Dulbecco's Modified Eagle's Medium (DMEM) supplemented with 7% fetal bovine serum (FBS) and 1% penicillin/streptomycin at 37°C in a 5% CO_2_ humidified incubator. Immortalized WT, Atg5^−/−^, Atg7^−/−^, MEKK1^−/−^ MEFs and MDA-MB-231 cells were maintained at 37°C in DMEM supplemented with 10% FBS and 1% penicillin/streptomycin, in a humidified incubator with an atmosphere containing 5% CO_2_. MDA-MB-231 cells that stably expressed shBeclin1 were generated by Lipofectamine 2000 transfection and geneticin selection. The cultured cells were transiently transfected using the calcium phosphate method or Lipofectamine 2000 (Invitrogen, Camarillo, CA, USA). To induce autophagy, we used PBS++ [8 g NaCl, 0.6 g KCl, 1.44 g Na_2_HPO_4_, 0.24 g KH_2_PO_4_, 100 mg MgCl_2_·6H_2_O, 100 mg MgSO_4_·7H_2_O, 350 mg NaHCO_3_, 185 mg CaCl_2_·2H_2_O per 1 L H_2_O, pH 7.1] for nutrient-deprivation [47].

### Luciferase reporter assay

The HEK293 cells, *Atg5*^+/+^, *Atg5*^−/−^, *MEKK1*^+/+^ and *MEKK1*^−/−^ MEFs were cotransfected with 4× CSL-Luc (a 4-time repeating section of the RBP-Jk target sequence, CGTGGGAA, with the luciferase gene) and β-galactosidase together with the indicated vector constructs. 48 h after transfection, the cells were lysed in chemiluminescent lysis buffer [18.3% of 1 M K_2_HPO_4_, 1.7% of 1 M KH_2_PO_4_, 1 mM phenylmethyl sulfonyl fluoride (PMSF), and 1 mM dithiothreitol (DTT)] and the luciferase assays were analyzed using a Luminometer. The luciferase reporter activity in each sample was normalized according to the β-galactosidase activity, which had been measured in the same sample [48].

### Real-time-qPCR analysis

Total RNA was prepared from cells using TRIzol reagent (MRC) according to the manufacturer's instructions. After quantification, cDNA was synthesized from 1 μg of RNA. Real-time quantitative PCR was performed using the Power SYBR Green PCR Master Mix (Applied Biosystems) in the Bio-rad iQ5 Quantitative PCR system. The primers used are as follows: human Notch1 (5′-TTGGGAGGAGCAGATTTTTG-3′ and 5′-CACTGGCATGACACACAACA-3′), human Hes1 (5′-AGGCTGGAGAGGCGGC TAAG-3′ and 5′-TGGA AGGTGACACTGCGTTGG-3′), (5′-GGTGCTGATAAC AGCGGAAT-3′ and 5′-TGAGCAAGTGCTGAGGGT TT-3′), human Hes5 (5′-GGTGCCTCCACTATGATC CTTA-3′ and 5′-TCCACGTGACTGAGAGTTCAA T-3′), human hes6 (5′-CGAAGTGCTGGAGCTGACGGTG-3′ and 5′-CACT GGATGTAGCCGGCAGCGAA-3′), human Hey1 (5′-GCATCTCAACAACTACGCTTCCCA-3′ and 5′-TGTGCGGGTGATGTCCGAA-3′), human Hey2 (5′-GGGTAAAGGCTACTTTGACGCAC-3′ and 5′-CG GAATCCTATGCTCATGAA-3′), human p21 (5′-GGCCC AGTGGACAGCGAGCA-3′ and 5′-CCCAGGCGAAGT CACCCTCC-3′), human p27 (5′-GGTTAGCGGAGC AATGCG-3′ and 5′-TCCACA GAACCGGCATTTG-3′), and human c-Myc (5′-TCCACAGAACCGGCATTTG-3′ and 5′-GCTGTGAGG AGGTTTGCTGTG-3′) [49].

### Immunoblot analysis

Immunoblotting analyses were carried out as described previously [19]. The cultured cells were lysed in 1 ml of radioimmunoprecipitation assay buffer [50 mM Tris-HCl (pH 7.5), 150 mM NaCl, 1% Nonidet P-40, 0.5% sodium deoxycholate, 0.1% SDS, 1 mM PMSF, 1 mM DTT, and 2 μg/ml each of leupeptin and aprotinin] for 30 min at 4°C. The cell lysates were then subjected to 15 min of centrifugation at 12,000 g at 4°C. The resultant soluble fraction was boiled in Laemmli buffer [63 mM Tris-HCl (pH 6.8), 10% Glycerol, 2% SDS, 5% β-mercaptoethanol, 0.0025% Bromophenol blue] and subjected to SDS-PAGE. After gel electrophoresis, the separated proteins were transferred via electroblotting onto polyvinylidene difluoride (PVDF) membranes. The membranes were then blocked with phosphate-buffered saline solution [137 mM NaCl, 2.7 mM KCl, 4.3 mM Na_2_HPO_4_, 1.4 mM KH_2_PO_4_, pH 7.4] containing 0.1% Tween 20 and 5% nonfat milk. The blotted proteins were then probed with anti-Notch1, anti-β-actin, anti-p62, anti-Fbw7 (Cdc4), anti-GFP, anti-LC3, anti-Myc, anti-HA, or anti-Flag, followed by incubation with anti-mouse horseradish peroxidase-conjugated secondary antibodies and anti-rabbit horseradish peroxidase-conjugated secondary antibodies. The blots were then developed using enhanced chemiluminescence (ECL).

### Co-immunoprecipitation assay

The cells were lysed in 1 ml of lysis buffer [20 mM Tris-HCl (pH 7.4), 2 mM EDTA, 25 mM NaF, 1% Triton X-100, 2 μg/ml each of leupeptin and aprotinin] for 30 min at 4°C. After centrifugation at 12,000 g for 15 min, the supernatants were subjected to immunoprecipitation with specific antibodies. After overnight incubation, Protein A agarose beads (GE healthcare) were added, and the samples were incubated for 1 h at 4°C on the rotator. The resulting immunoprecipitates were washed three times with PBS, and the immune complexes were eluted with Laemmli buffer for 5 min at 95°C and visualized by immunoblotting.

### *In vitro* kinase assay

To analyze the MEKK1 kinase activity, cells were harvested and lysed in lysis buffer [50]. After centrifugation at 12,000 g for 15 min at 4°C, the supernatants were subjected to immunoprecipitation with specific antibodies. The immune complexes were then coupled with protein A-agarose for 1 h at 4°C, after which they were pelleted through centrifugation and rinsed three times with lysis buffer. The kinase reaction was performed through the incubation of the immunopellets for 1 h at 30°C with 2 μg of substrate proteins in 20 μl reaction buffer containing 0.2 mM sodium orthovanadate, 10 mM MgCl_2_, 2 μCi [γ^32^P] ATP, 20 mM HEPES, and pH 7.4. The phosphorylated substrates were then visualized through SDS-PAGE and quantified using a Fuji BAS 2500 phosphoImager.

### Immunofluorescence staining

HEK293 cells were seeded onto coverslips in 6-well culture plates. Cells were washed with PBS and fixed by 4% paraformaldehyde for 15 min at room temperature. Cells were then washed three times with PBS and permeabilized with 1% BSA in PBS solution with 0.1% Triton X-100 at 4°C for 5 min. Cells were blocked with 1% BSA in PBS for 30 min. Thereafter, cells were incubated with specified primary and secondary antibodies for 1 h respectively with three washes in between. The primary antibodies against p62 and Notch1-IC V1744 were diluted in 1% BSA/PBS. The fluorescent-conjugated secondary antibodies were anti-mouse Alexa Fluor 488, anti-rabbit Alexa Fluor 488, and anti-rabbit Alexa Fluor 532 and were diluted in 1% BSA/PBS. The nuclei were stained with ToPro3. Cells were mounted with anti-fade solution and visualized using confocal microscopy with LAS AF software (Leica). Scale bars represent 25 μm as indicated.

### Subcellular fractionation

Forty-eight hours after transfection, HEK293 cells were rinsed with ice-cold PBS before resuspension in ice-cold buffer A (10 mM HEPES at pH7.9, 10 mM KCl, 1.5 mM MgCl_2_, 0.5 mM DTT, 0.05% Nonidet P-40, and 2 μg/mL each of leupeptin and aprotinin). After 15 min on ice, the dispersed cells were harvested by centrifugation for 10 min at 720 g and 4°C to separate the cytoplasm from the nuclear component. The resultant supernatant was then used as the cytosolic fraction. The pellet was homogenized with buffer B (5 mM HEPES at pH 7.9, 0.3 M NaCl, 1.5 mM MgCl_2_, 0.2 mM EDTA, 0.5 mM DTT, and 26% glycerol). The pellet was lysed, and nuclear proteins were released by sonication on ice. After 30 min on ice, the homogenates were centrifuged for 20 min at 12,000 g and 4°C. The resultant supernatants were then used as the nuclear fractions. The nuclear and cytosolic fractions were quantified by using Bradford assay, and 20 μg of each fraction was analyzed by immunoblotting.

### Wound healing assay

MDA-MB-231 cells were plated into 6-well plates at 5 × 10^5^ cells per well. A scratch was made on a uniform layer of cells using a sterile micropipette tip and cells were washed to remove debris [51]. Photographs of the same area of the wound were taken after 8, 16, and 24 h to measure the width of the wound. To evaluate the effect of Notch1 inhibitors on the cell migration, cells were pretreated with 2 μM DAPT for 6 h prior to seeding in the plates. Assays were done in triplicate with excellent reproducibility.

### Migration assay

The migration assay was performed using Transwell inserts (Corning) with an 8 μm pore polycarbonate membrane. The upper side of the Transwell membrane was coated with 20 μg/ml fibronectin. DMEM supplemented with 10% FBS was added to the bottom chamber of the transwell plate. MDA-MB-231 cells were seed into the upper chamber at 10^4^ cells per well. After allowing cell migration for 16 h, non-migratory cells were removed from the upper side of the membranes using cotton swabs. Migratory cells on the lower side of the membranes were washed with PBS, fixed with 4% paraformaldehyde for 15 min, and stained with 0.01% DAPI for 3 min. Migratory cells were determined by fluorescence microscopy (Leica) and the number of cells in three random fields were counted. To evaluate the effect of Notch1 inhibitors on cell migration and invasion, cells were pretreated with 2 μM DAPT for 6 h prior to loading onto the migration chamber. Assays were done in triplicate with excellent reproducibility.

### Matrigel invasion assay

For invasion assays, MDA-MB-231 cell invasion was measured using the Biocoat Matrigel Invasion Chamber (BD bioscience) with 8 μm pore size according to manufacturer's protocol. 10% FBS DMEM was added to the bottom chamber of the transwell plate. Cells were plated in the upper chamber at 10^4^ cells per well. MDA-MB-231 cells were allowed to migrate for 16 hr. After the incubation period, non-invading cells on the upper side of the filter were gently removed from the upper chamber with cotton-tipped swabs. The cells that had invaded the lower surface of the filter were washed with PBS, fixed with 4% paraformaldehyde, and stained with DAPI. The number of invasive cells from three randomly selected fields was counted using a fluorescence microscope. To evaluate the effect of Notch1 inhibitors on cell migration and invasion, cells were pretreated with 2 μM DAPT for 6 h prior to loading onto the invasion chamber. Assays were done in triplicate with excellent reproducibility.

### Colony formation assay

For the clonogenic assay, MDA-MB-231 cells were plated in 24-well plates at 100 cells per well. Cells were incubated for 10 days and the medium was replaced with fresh medium every three days. After 10 days, colonies were fixed with 4% paraformaldehyde for 15 min, stained with 0.05% crystal violet, and counted. Soft agar assays were performed by coating 6-well tissue-culture plates with 2 ml of 0.7% Noble agar/growth media (10% FBS/DMEM, 2 μg/ml puromycin) and allowed to solidify at 20°C. MDA-MB-231 cells (5 × 10^3^) were plated in the top layer of 1.5 ml of 0.3% Noble agar/growth media. Growth media was added every 3 days. Cells were stained with 0.05% crystal violet, destained with sterile water and counted 28 days after seeding. To evaluate the effect of Notch1 inhibitors on the colony formation ability, cells were pretreated with 2 μM DAPT for 6 h prior to seeding in the plates [33]. Assays were done in triplicate with excellent reproducibility.

### Human tissue samples

All of the human tissue samples were obtained from individuals undergoing breast tumor resection at the Chonnam National University Hwasun Hospital National Biobank of Korea, which is supported by the Ministry of Health, Welfare and Family Affairs. Expression levels were analyzed by immunoblotting, quantified using ImageJ, and was normalized to β-actin levels.

### Statistics

Data represent the mean ± SD for three independent experiments. Statistical significance was analyzed by a two-tailed Student's *t* test using the SigmaPlot 11.0 software. P < 0.05 was considered statistically significant.

## SUPPLEMENTARY MATERIALS


