# Supplementary material for: Autophagy negatively regulates tumor cell proliferation through phosphorylation dependent degradation of the Notch1 intracellular domain
Source: Oncotarget. 2016 Oct 27;7(48):79047–63. doi: 10.18632/oncotarget.12986 (PMC5346697; doi:10.18632/oncotarget.12986)
Supplement: Supplementary file 1 [file oncotarget-07-79047-s001.pdf]

Autophagy negatively regulates tumor cell proliferation through phosphorylation dependent degradation of the Notch1 intracellular domain

Supplementary Materials

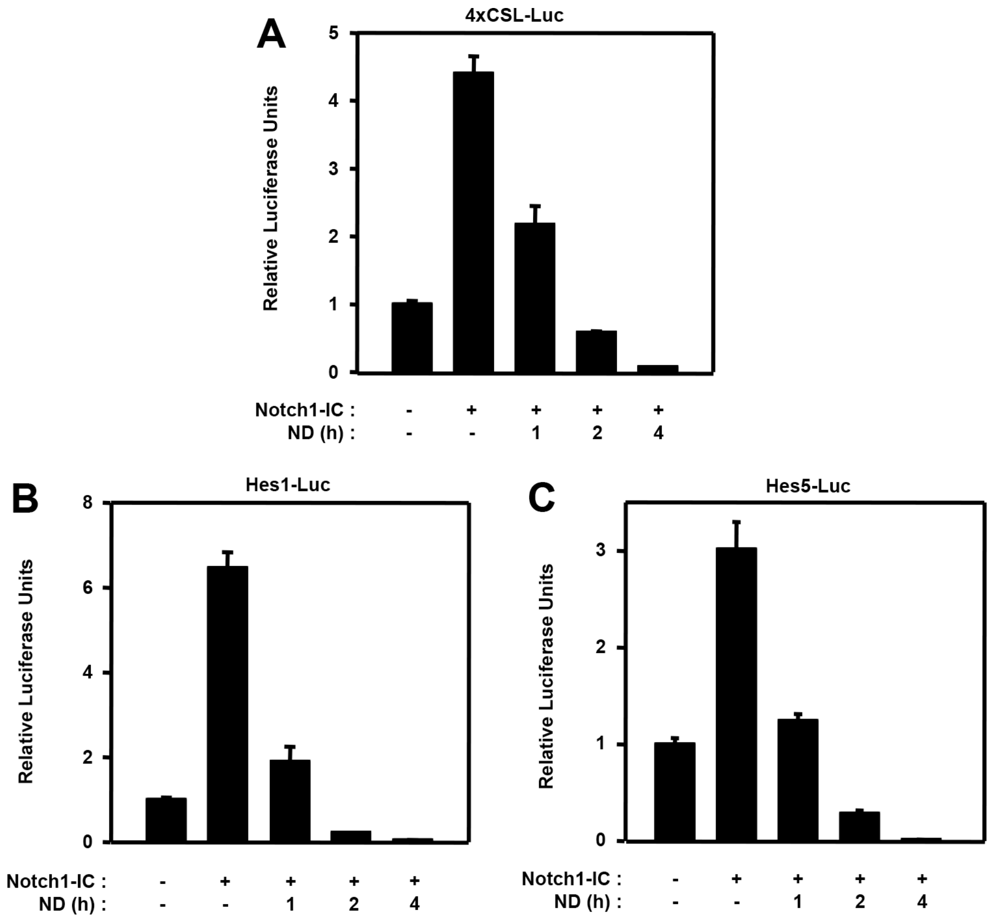

Supplementary Figure S1: Time-dependent starvation attenuates the transcriptional activity of Notch1-IC. **(A)** Time-dependent starvation reduces the transcription of 4xCSL-luc. HEK293 cells were transfected with 4xCSL-Luc, together with the indicated plasmids. 48 h after transfection, the cells were analyzed for Notch1-IC transcriptional activity (fold induction). **(B)** Time-dependent starvation reduces the transcription of Hes1-Luc. HEK293 cells were transfected with the Hes1-Luc, together with the indicated plasmids. 48 h after transfection, the cells were analyzed for Notch1-IC transcriptional activity (fold induction). **(C)** Time-dependent starvation reduces the transcription of Hes5-Luc. HEK293 cells were transfected with the Hes5-Luc, together with the indicated plasmids. 48 h after transfection, the cells were analyzed for Notch1-IC transcriptional activity (fold induction).

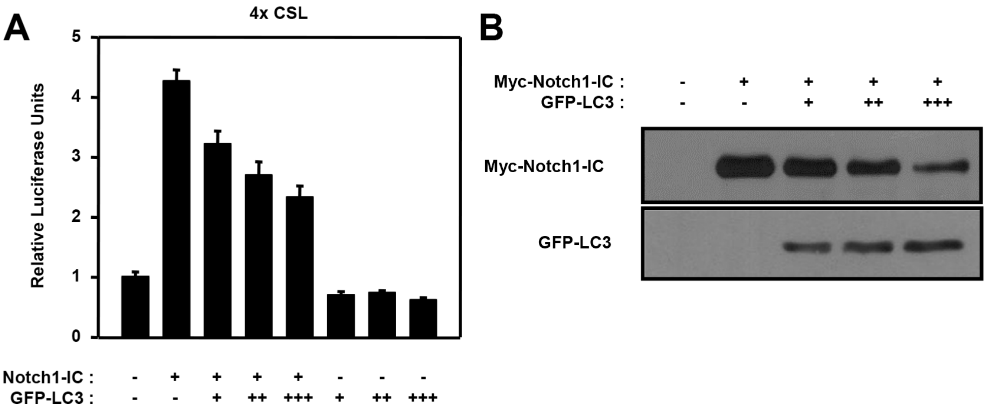

Supplementary Figure S2: LC3 negatively regulates Notch signaling. (**A**) LC3 reduces the transcriptional activity of Notch1-IC. HEK293 cells were transfected with 4xCSL-Luc, together with the indicated plasmids. 48 h after transfection, the cells were analyzed for Notch1-IC transcriptional activity (fold induction). (**B**) LC3 reduces the Notch1-IC protein level. HEK293 cells were transfected with the indicated plasmids. 48 h after transfection, the cells were subjected to immunoblotting using anti-Myc and anti-GFP antibodies. Results are representative of at least 3 independent experiments.

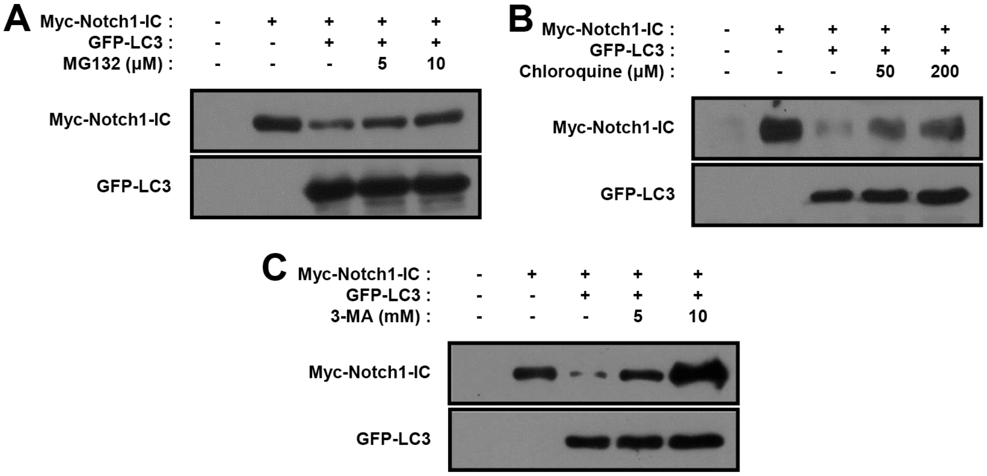

Supplementary Figure S3: Notch1-IC is degraded by various pathways. (**A**) Notch1-IC was degraded by LC3 through the proteasome-dependent pathway. HEK293 cells with the indicated plasmids underwent treatment with MG132 for 6 h or no treatment and were subjected to immunoblotting using anti-Myc and anti-GFP antibodies. (**B**) Notch1-IC was degraded by LC3 through the lysosomal pathway. HEK293 cells with the indicated plasmids underwent treatment with chloroquine for 6 h or no treatment and were subjected to immunoblotting using anti-Myc and anti-GFP antibodies. (**C**) Notch1-IC was degraded by LC3 through the autophagosomal pathway. HEK293 cells with the indicated plasmids underwent treatment with 3-MA for 6 h or no treatment and were subjected to immunoblotting using anti-Myc and anti-GFP antibodies. Results are representative of at least 3 independent experiments.

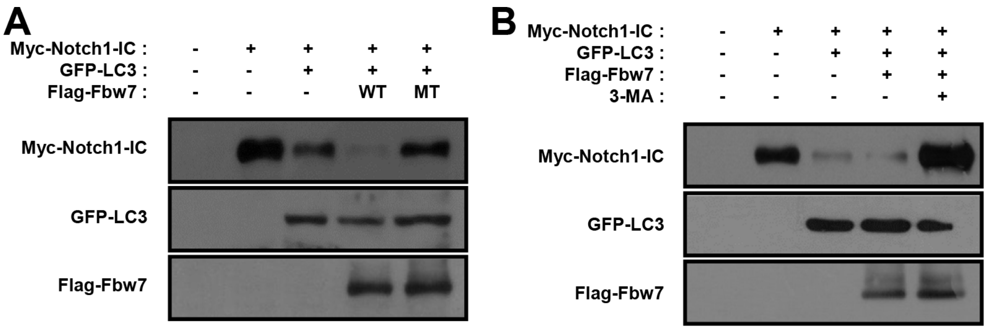

Supplementary Figure S4: Fbw7 is crucial for the degradation of Notch1-IC by LC3. (**A**) Notch1-IC degradation by LC3 requires the E3 ligase activity of Fbw7. HEK293 cells with the indicated plasmids were subjected to immunoblotting using anti-Myc, anti-GFP, and anti-Flag antibodies. (**B**) Fbw7-dependent Notch1-IC degradation by LC3 proceeded via the autophagic pathway. HEK293 cells with the indicated plasmids underwent treatment with 10 mM 3-MA for 6 h or no treatment and were subjected to immunoblotting using anti-Myc, anti-GFP, and anti-Flag antibodies. Results are representative of at least 3 independent experiments.
